# Multifunctional Sol–Gel Coatings for Both Anticorrosion and Electrical Conduction Properties

**DOI:** 10.3390/ma18092011

**Published:** 2025-04-29

**Authors:** Clément Genet, Hiba Azougaghe, Edouard Bréniaux, Robin Montpellaz, Marie Gressier, Florence Ansart, Olivier Gavard, Marie-Joëlle Menu

**Affiliations:** 1 Centre Interuniversitaire de Recherche et d’Ingénierie des Matériaux (CIRIMAT), Université Toulouse 3 Paul Sabatier, Toulouse INP, CNRS, Université de Toulouse, 118 Route de Narbonne, 31062 Toulouse cedex 9, France; clement.genet@amphenol-socapex.fr (C.G.);; 2 Amphenol Socapex, 948 Promenade de l’Arve, BP 29, 74300 Thyez, France

**Keywords:** organic–inorganic coatings, sol–gel process, anticorrosion, electrical conduction, carbon fillers

## Abstract

This work is part of a current and essential issue aiming to find a solution for the replacement of chromium(VI) and cadmium in the surface treatment process applied to electrical connectors. The application of a protective coating obtained by the sol–gel route proves to be an interesting alternative method and numerous studies describe efficient anticorrosion coatings to protect various metallic alloys. The issue of electrical connectors made of 6061 alloy is to combine anticorrosion protection and electrical conduction, which are antagonistic properties, so multifunctional sol–gel coatings and/or architectures have to be synthesized and shaped on connectors. In this work, several experimental parameters, such as the type of carbon filler, the hydrolysis ratio, the precursors’ introduction order are studied and evaluated to achieve industrial requirements. Thus, aqueous suspensions of carbon fillers have been introduced into sol–gel formulations to give rise to conductive coatings (200–500 mΩ_□_) with high anticorrosion properties (500 h NSS resistance), in which thickness is less than 10 microns. The incorporation of organic additives poly(2-ethyl-2-oxazoline) or hydroxypropylmethylcellulose positively impacts the flash point of the sol (>60 °C) making the sol–gel process compatible with the HSE recommendation and the ATEX standard.

## 1. Introduction

**Figure 1:**
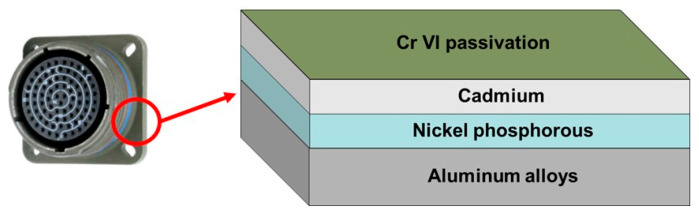
Actual standard coating for an electrical connector in agreement with the MIL-DTL-38999 (Amphenol Socapex, Thyez, France).

Over the last several years, the industrial sector is undergoing a real change in its processes with the implementation of new European regulations, REACH, and RoHS, and Cr(VI) and Cd are one of the targeted elements [1]. Cr(VI) is used in many formulations and processes to improve material properties, such as corrosion resistance or mechanical durability. In the field of industrial connectors, one application of Cr(VI) passivated cadmium compounds is the manufacture of protective multifunctional coatings (Figure 1). These coatings are applied in complex shape parts to both protecting the connectors against corrosion and maintaining their electrical properties. These connectors must be resistant to harsh environments, a corrosive atmosphere, and provide an electromagnetic shield in working conditions. Cr(VI)-based treatment is employed as a passivation layer to avoid a chemical reaction with the cadmium compound. Cr(VI)-passivated cadmium brings, at the same time, a specific coloration and improve the corrosion resistance of the architectural coating without decreasing the electrical performances. Since 2017, the ban has been in effect, and connector manufacturers obtain authorization from ECHA only if alternative solutions are being studied.

Among the solutions studied, the sol–gel process is known as an alternative method for the development of new multifunctional coatings [2,3].

Hybrid coatings, combining organic and inorganic parts obtained by the sol–gel method, offer several advantages: flexibility, corrosion resistance, mechanical performance, and the capability of modulating the sol–gel formulation to achieve new properties [4,5,6]. The specifications of electrical connectors are strict; the coating obtained by the sol–gel process must ensure numerous functional specifications, such as anticorrosion protection with a resistance greater than 500 h at NSS (neutral salt spray), electrical conductivity, which must be maintained (electrical resistance < 200 mΩ_□_), but also second level properties, such as mechanical resistance, dark and non-reflective color, etc.; all of these properties must be combined in a coating whose thickness must not exceed 20 µm. This paper will explore the possibility of combining the two main properties within a single coating, which, to our knowledge, has never been described, probably because these two properties may appear difficult to match: electrical conduction and anticorrosion protection.

A previous work [7,8] presents a state-of-the-art on the subject and demonstrates the possibility of developing a sol–gel formulation containing carbon fillers to ensure electrical conductivity but the coatings obtained are not sufficiently protective against corrosion. The coating obtained after the dip-coating process and optimized thermal treatment exhibited a corrosion resistance to NSS of 168 h (on model specimen) and an electrical resistance of 150 mΩ_□_ (measured by the two probes method for electrical surface resistance characterization). It is, therefore, essential to improve corrosion protection to meet specifications and achieve 500 h of neutral salt spray resistance, as well as to improve the robustness of the coating development process to improve the reproducibility of their performance and their durability. In addition, considering the transfer from laboratory to industrial scale, various parameters must also be considered for the application and safety of the process. For example, during sol preparation, the use of nanometric carbon fillers is a point of vigilance and the HSE (Health Safety Environnement) recommendation aims to eliminate this use as much as possible for the protection of employees and the environment. In addition, the use of a large volume of sols can be problematic, especially in a surface treatment department using large tanks, due to the production of alcohol during the hydrolysis and condensation reactions of the sol–gel process. These working conditions require control of the potential explosive atmosphere above the tanks, governed by the ATEX standard, if the flash point of the sols is not sufficiently high [9]. In this work, three aqueous suspensions of carbon-based fillers will be evaluated; the introduction of additives to increase the flash point of the sol–gel formulations will also be studied in order to provide a response to the industrial problem linked to HSE recommendation and the ATEX standard.

Multifunctional organic–inorganic sol–gel coatings are investigated involving 3-glycidyloxypropyl)trimethoxysilane (GPTMS) and zirconium (IV) propoxide (TPOZ) precursors in the presence of water and alcohol to provide the organic–inorganic network responsible for the protective barrier effect. The study will begin by selecting the carbon filler and evaluating the properties of the sols and coatings. The second part is dedicated to assessing the effect of the filler concentration on the sols and coatings. For each part, different parameters of the sol formulation are varied, such as filler mixture, filler proportions, and hydrolysis ratio (H). The effect on the viscosity and the flash point (FP) are evaluated. The last part is focused on the improvement of the sol’s properties (the target parameters are a viscosity higher than 10 mPa·s and a flash point higher than 60 °C) and coating performances (corrosion resistance and electrical conduction) by adding organic additives in a perspective of industrial transfer.

## 2. Materials and Methods

### 2.1. Substrates and Process

The studied substrate is an aluminum alloy AA6061, and its composition is given in Table 1. Sample dimensions are L = 80 mm, l = 40 mm, and e = 1 mm. Then, a NiP (high phosphorous content, 10–13%) layer of 20 µm is deposited by an electroless process. Before the sol–gel deposition, an activation of the NiP is performed with a degreasing bath based on Alumal Clean 101 (50 °C-10 min) and an activation bath based on Metex 659 (30 °C-10 min).

The sols are deposited on the substrate by dip-coating with Bungard RDC21-K (Bungard Elektronik GmbH & Co. KG, Windeck, Germany) equipment. The withdrawal speed can be controlled between 50 to 1000 mm min^−1^. A withdrawal speed of 200 mm min^−1^ was used if nothing is specified for the results reported in this work. After deposition, the coatings were consolidated by thermal treatment in air using an oven Memmert UN30 Plus (Memmert, Schwabach, Germany) with two ramps of 60 min to 60 °C and 120 min to 120 °C.

### 2.2. Precursors and Sol–Gel Formulations

**Figure 2:**
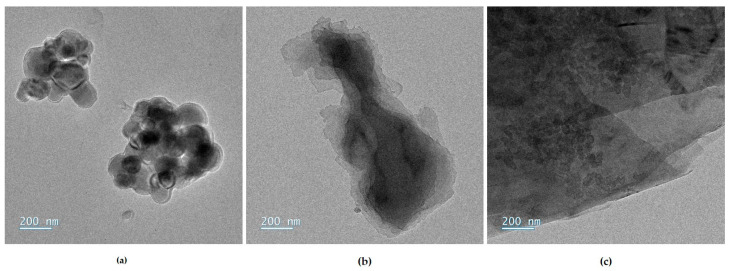
MET pictures of carbon fillers from IMERYS with (**a**) carbon black suspension F1, (**b**) graphite suspension F2, and (**c**) carbon black–graphite mixture suspension F3.

Sols were prepared from the following precursors: (3-glycidyloxypropyl)trimethoxysilane (GPTMS, ≥98%, Sigma Aldrich, St. Louis, MO, USA), and zirconium(IV) propoxide (TPOZ, 70 wt% in 1-propanol, Sigma Aldrich). The precursors are mixing together with glacial acetic acid (VWR Chemicals, Radnor, PA, USA). Three carbon-based fillers as aqueous suspensions, provided by Imerys, were used and presented in Figure 2. The filler suspensions, noted FX, contain different morphologies of carbon, F1 is based on carbon black of around 100 nm, F2 is a graphite sheets suspension, and F3 is a mix of 50–60 nm sized carbon black particles (2/3 wt%.) and large graphite sheets (1/3 wt%.). Organic additives introduced in the sol formulations are given in Table 2; they were chosen for their high boiling point and high flash point. No data are found for the oxazoline derivative (AZ). The subscript number to the right of the abbreviation indicates the molar mass of the polymer.

The formulations are denominated GZ_x_-yF, with G and Z for the GPTMS and TPOZ, respectively, x for the hydrolysis ratio defined as n(H_2_O)/(3nGPTMS + 4nTPOZ), y for the m_cond_/m_insu_ ratio, where m_cond_ is the mass of fillers providing the electrical conductivity of the coating and m_insu_ is the mass of sol–gel precursors which produces the insulating part of the coating, F for the reference of the carbon-based fillers.

Sols were prepared according to the protocol detailed below for the GZ_50_-90F1 formulation; the preparation was similar for the other compositions. For the GZ_50_-90F1 formulation, GPTMS (3.62 g, 0.0153 mol) and TPOZ (3.51 g, 0.0075 mol) precursors were mixed together with acetic acid (1.80 g, 0.030 mol) and stirred for 15 min for homogenization. Then, this mixture was added to an aqueous suspension of the conductive fillers (22.91 g, 0.53 mol of C) previously diluted in water (51 g, 2.81 mol); the final mixture was maintained under magnetic stirring for about 1 h. After that, the stirring of the sol was stopped, and the sol was then aged over 24 h at room temperature in a closed flask.

When the sol contained a mixture of carbon fillers, the suspensions were premixed before the introduction of the sol–gel precursors. In the case of formulations containing an additive, it was previously dissolved in water (a few minutes for PE, PEG; 30 min for PEG, PVA, PVP; 5 h for HPMC); then, the carbon fillers were added; the suspension was mixed for an additional 30 min; then, the sol–gel precursors were added. The sol was stirred for 1 h and then left for aging.

### 2.3. Characterization Techniques

- Sol characterization

The viscosity was measured by the Rheomat RM 100 rheometer (Lamy Rheology, Champagne-au-Mont-d’Or, France), for three shearing rates (322, 644 and 966 s^−1^). Three measurements were made for each speed, and the result is an average of these values.

The flash point of the sols is measured using the Seta Flash series 3E active cool closed cup tester (Stanhope-Seta, Surrey, UK). A small volume of the sol was taken (4 mL) and heated in the equipment. The temperature was aligned at 30 °C and increased by increments of 1 °C. The flash point was determined when a flash happens and the retained value was an average of three measurements.

- Coating characterization

Electrical properties were characterized by the surface resistance measured with the multimeter Keithley 2110 51/2 (Tektronix, Beaverton, OR, USA) by the two probes method [10]. The measure was made on each face of the sample in three different areas to obtain an average value. The microstructure of the fillers was characterized by TEM JEOL JEM 1011 (JEOL, Tokyo, Japan) and the architecture of the coatings was characterized by SEM-FIB FEI HELIOS 600i-EDS (Thermo Fisher Scientific, Waltham, MA, USA). Coating thicknesses were measured by SEM observation after FIB preparation. The anticorrosive barrier properties of the coatings were evaluated by NSS exposure in a Q-Fog SSP600 (Labomat Essor, Saint Denis, France) dedicated to the corrosion ageing. The samples were exposed for 500 h with a visual observation at regular intervals, every 24 h during the first week, and then every week. A duration of 500 h of exposure without any full-face degradation of the coating (corrosion puncture) was considered to be a compliant performance.

## 3. Results and Discussion

In this paragraph, after presenting and selecting the type of carbon fillers, sols are formulated, varying on the following:

(i) Firstly, proportions and/or concentrations of carbon fillers for a hydrolysis ratio equal to 50 associated to the corresponding sols characteristics and coatings morphologies and properties;
(ii) Then, the hydrolysis ratio and the incorporation of organic additives in order to modify the viscosity and the flash point of the sols.

### 3.1. Carbon Filler’s Selection

For this first part, the F1 and F2 suspensions are used to introduce various proportions of the carbon black and graphite in the formulations. The aim here is to understand the contribution of each carbon filler to converge towards an optimized formulation. Considering the previous work [7] H the hydrolysis ratio is set at 50; it corresponds to the water amount relative to the sol–gel precursors quantity. The filler amount is fixed at 0.9, corresponding to the mass ratio between the carbon fillers and sol–gel precursors in the coating, or as the expected ratio between the conductive part and insulating part in the coating (m_cond_/m_insu_). This value varies, considering different proportions of carbon black and graphite but keeping the same proportion of the conductive part expected in the coating. All the formulations are gathered in Table 3 with the sol characteristics.

The flash points (FPs) of the sols are similar and in the range between 51 and 53 °C; these values are higher than those measured for a GZ_50_ sol without carbon fillers (38 °C). So, the presence of a carbon filler allows to increase the FP but the F1-F2 filler proportion has no real impact on the FP. The viscosity slightly decreases when the graphite is the main filler; the improvement of the sol flow may be attributed to the morphology of the F2 graphite nanoparticles [11,12].

**Figure 3:**
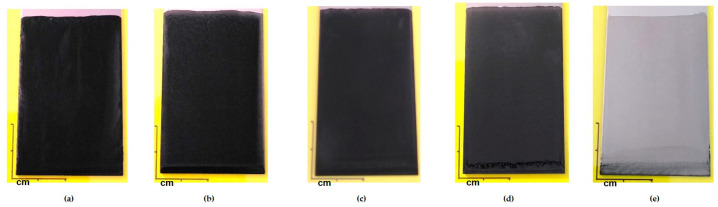
Photographs of the coatings with a different proportion of carbon black and graphite particles: (**a**) GZ_50_-90F1, (**b**) GZ_50_-60F1-30F2, (**c**) GZ_50_-45F1-45F2, (**d**) GZ_50_-30F1-60F2, and (**e**) GZ_50_-90F2.

Deposition of the sols by dip-coating using a withdrawal speed of 600 mm min^−1^ provide covering coatings. The aspect of coatings with variations in the carbon fillers (F1 and F2) is analyzed; Figure 3. At the macroscopic scale, the coatings mainly composed with the carbon black exhibit a dark and a non-reflective coloration. When the proportion of graphite particles increases, the appearance tends towards a grey coloration. Finally, the coating with only graphite filler is translucent.

**Figure 4:**
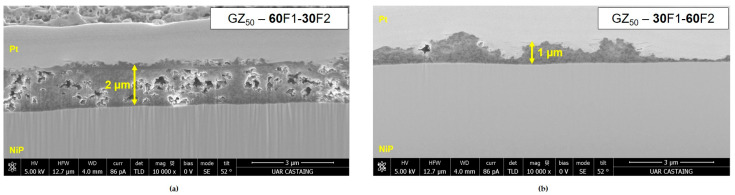
SEM pictures in cross-section after FIB preparation for (**a**) GZ_50_-60F1-30F2 coating and (**b**) GZ_50_-30F1-60F2 coating.

Microstructural characterization was performed by scanning electron microscopy (SEM) with focused ion beam (FIB) preparation. The coatings containing a mixture of carbon black and graphite in a proportion of 60F1-30F2 and 30F1-60F2, respectively, are presented in Figure 4. The coating composed with the higher content of graphite exhibits an irregular thickness in the area observed (around 1 µm). For the coating with higher carbon black, the thickness is more homogenous and around 2 µm. There is also porosity dispersed into the volume. This difference can be explained by the graphite proportion and its impact on the sol viscosity. With a lower sol viscosity, the thickness is thinner for the same withdrawal speed. The graphite is known to confer a hydrophobic effect and induces a bad grip during the withdrawal. It can be the explanation of these heterogeneities in the final microstructure of the coating [13].

The different characteristics of the coatings, thickness, electrical resistance, and NSS resistance, are compiled in Table 3. Except for GZ_50_-90F2, the electrical resistance is almost the same for all the coatings, around 100 and 200 mΩ_□_. This range corresponds to the electrical resistance of the metallic substrate without a coating. So, the coatings based on carbon fillers preserve the electrical conduction of the system. The coatings with only the graphite have a higher resistance because of the heterogeneity of the coatings and the difficulty to achieve the electrical percolation threshold. For corrosion resistance, almost all the coatings have the same performance with a duration of 500 h with no pitting after NSS exposure. Only some partial defects appear after 336 h in NSS for the GZ_50_-30F1-60F2 and GZ_50_-90F2 coatings. A minimum of graphite is necessary to improve the electrical conductivity and the corrosion resistance of the coatings. Furthermore, a higher graphite proportion affects the homogeneity and thickness of the coatings. A threshold of 0.3 graphite F2 seems to be relevant for these formulations.

### 3.2. Filler’s Concentration Variation

Considering the previous results, the combination of carbon black and graphite seems clearly beneficial and promising. That is why, in this second part, we directly consider a commercial mixture, named the filler F3, corresponding to a mixture of carbon black (2/3 wt%.) and graphite (1/3 wt%.). The formulations with F3 are roughly similar to the formulation combining F1 and F2 fillers in GZ_50_-60F1-30F2. Nevertheless, it is necessary to determine the influence of the quantity of the filler on the properties of both sols and coatings. To do this, the formulation GZ_50_-100F3 is used as a reference. The ratio m_cond_/m_insu_ varied from 0.9 to 4. All formulations are gathered in Table 4, with the sol properties (viscosity and flash point).

**Figure 5:**
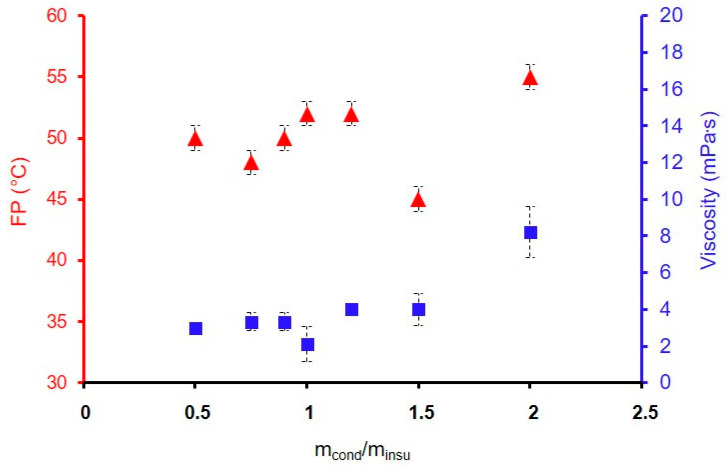
Influence of the ratio m_cond_/m_insu_ on the FP (▲) and the viscosity (■) for the formulations GZ_50_-yF3.

In Figure 5, the FP and the viscosity are represented as a function of the ratio m_cond_/m_insu_. The viscosity is relatively weak, with values in the range 2 to 4 mPa·s; for the ratio m_cond_/m_insu_, between 0.5 and 1.5. For a m_cond_/m_insu_ of 2, the viscosity increases to 8 mPa·s. For a ratio higher than 4 (not represented), the sol is too viscous, as a putty so the formulations are unsuitable. The FPs are in the range 48 and 55 °C for all formulations indicating that variation in the F3 ratio has no real impact on the FP. Only a weak rise is visible for the ratio 2 (55 °C compared to 52, 50, and 48 °C for other ratios). Here, the effect caused by the presence of carbon fillers on the flash point, whatever their proportion or nature, is once again demonstrated. When the hydrolysis ratio is increased to 75, the flash point of the formulation reaches 58 °C for a m_cond_/m_insu_ concentration between 0.9 and 1, which once again highlights the influence of the hydrolysis ratio on the flash point value. So, the FPs have similar values close to the target value of 60 °C.

**Figure 6:**
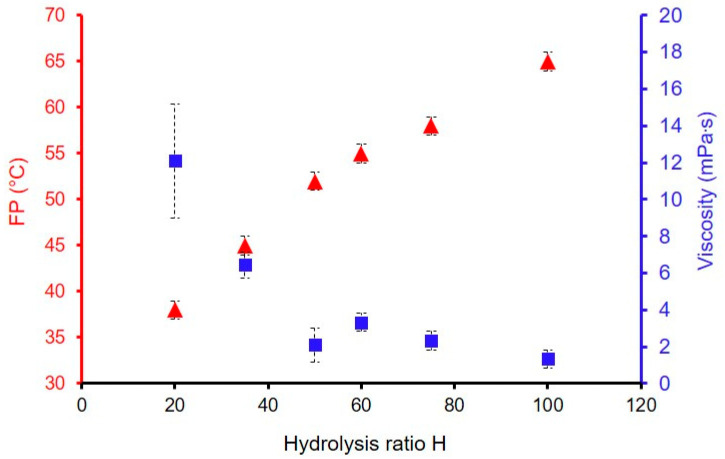
Influence of the hydrolysis ratio on the FP (▲) and viscosity (■) for the formulations GZ_x_-100F3.

The hydrolysis ratio H is evaluated from 12 to 100 for the formulation GZ_x_-100F3. The sol characteristics are presented in Table 5. For the formulation GZ_12_-100F3, the sol was too putty for the characterization and was not deposited. Figure 6 shows the influence of the hydrolysis ratio on the FP and the viscosity. The FP is higher when the H increases, with a maximum of 65 °C for the formulation GZ_100_-100F3 (up to the security value of 60 °C). This effect can be attributed to the reduction in the alcohol/water ratio, the alcohol being generated during the transformation of the sol-coating, or the FP measurement.

As expected, the viscosity increases when water amount is decreasing; this is in agreement with the values obtained for the lower hydrolysis ratios H = 20 and 35. It is, therefore, necessary to find a compromise between a viscosity that is not too high and a flash point sufficiently close to 60 °C. Such case can be found for the GZ_50_-100F3. This combined study of the fillers selection and concentration, and the hydrolysis ratio, is not sufficient to allow the development of the sols with a viscosity higher than 10 mPa·s and with a FP ≥ 60 °C.

**Figure 7:**
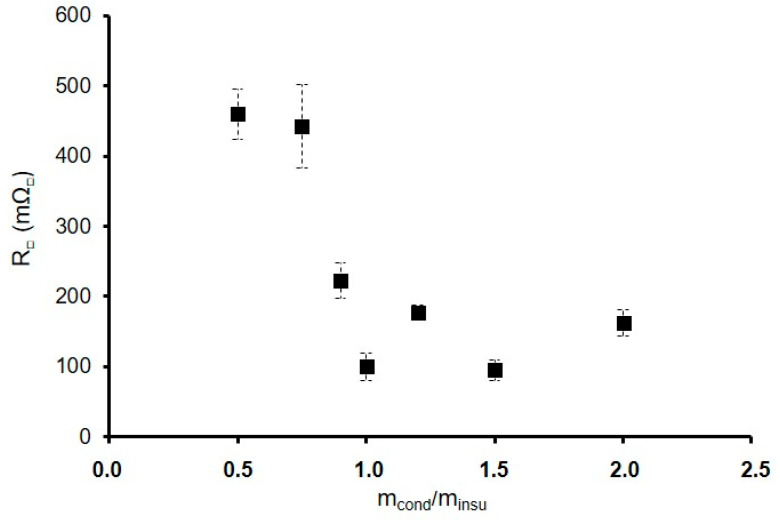
Evolution of the electrical resistance (■) for the formulations GZ_50_-m_cond_/m_insu_F3.

The study of the variation in the ratio m_cond_/m_insu_ can be useful to understand the electrical behavior evolution. The electrical resistance variation as a function of the ratio m_cond_/m_insu_ is represented in Figure 7. The electrical resistance stabilizes around 200 mΩ_□_ when the m_cond_/m_insu_ ratio reaches 0.9. There is an electrical percolation effect, with a transition between 460 mΩ_□_ to 100 mΩ_□_. The percolation appears just after the m_cond_/m_insu_ ratio of 0.75.

**Figure 8:**
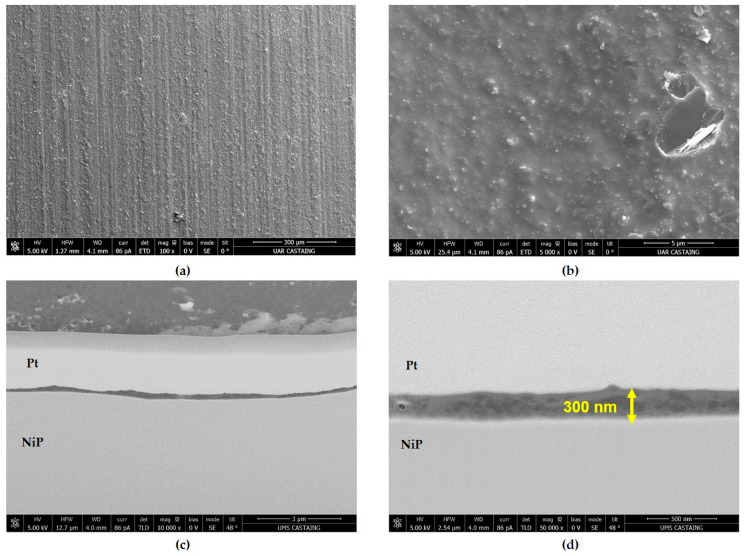
SEM pictures of the surface (**a**,**b**) and of the cross-section (**c**,**d**) after FIB preparation for the GZ_50_-100F3 coating (withdrawal speed of 200 mm min^−1^).

The electrical resistance of coatings was investigated with a variation in the hydrolysis ratio for formulations GZ_x_-100F3 listed in Table 5. The coatings GZ_20_-100F3 and GZ_100_-100F3 were too heterogeneous for the electrical resistance evaluation. For the other coatings the resistance is in the range 100–200 mΩ_□_, so the hydrolysis ratio has no influence on it. For corrosion resistance, the coatings have the same performance with a resistance of 500 h with no pitting after NSS exposure. The microstructural characterization performed by scanning electron microscopy (SEM) shows covering and homogeneous coatings, but the thickness is less than 500 nm. For example, Figure 8 presents the surface and the cross-section views for the GZ50-100F3. On the micrograph of the coating surface (Figure 8a,b), the low thickness of the coating still reveals the morphology of the substrate. At higher magnification, carbon fillers embedded in the sol–gel matrix are clearly visible. These observations are endorsed in the cross-section micrographs (Figure 8c,d), which show a thin, but homogeneous covering coating in the range 100–300 nm. Carbon nanoparticles are also visible in the sol–gel matrix all along the thickness of the coating.

Compared to the initial work [7], the main positive result with these new coatings is the formation of a coating in a monolayer application with convenient anticorrosion and electrical conduction properties. However, their thickness is too small for the connector’s durability in harsh environments.

### 3.3. Properties’ Improvement and Potential Industrial Transfer

Aiming to improve the sol viscosity for the development of robust coatings, and to increase the flash point for security reasons, different additives can be added during the formulation. In the literature, several organic additives are listed as potential candidates for these applications [14,15,16]. Five additives have been evaluated and introduced at different mass percentages (mass total of the sol) with a first dissolution step in water. The percentage added varies between 1 to 30% depending on the additives.

In this study, the starting formulation is GZ_75_-90F3. With additives, the sol formulation name is GZ_75_-90F3-zADD with z being the mass percentage of the additives into the sol, and ADD being the additive chemical nature. The organic additives used are ethylene glycol (EG), polyvinyl alcohol Mw = 13,000 (PVA), polyethylene glycol Mw = 1500 (PEG), polyvinyl-pyrrolidone Mw = 3500 (PVP), poly(2-ethyl-2-oxazoline) Mw = 500 (AZ), and hydroxypropylmethylcellulose Mw = 1260 (HPMC). The ratio m_cond_/m_insu_ is fixed at 0.9 and the hydrolysis ratio is equal to 75. Characteristics of the sols and coatings are reported in Table 6.

**Figure 9:**
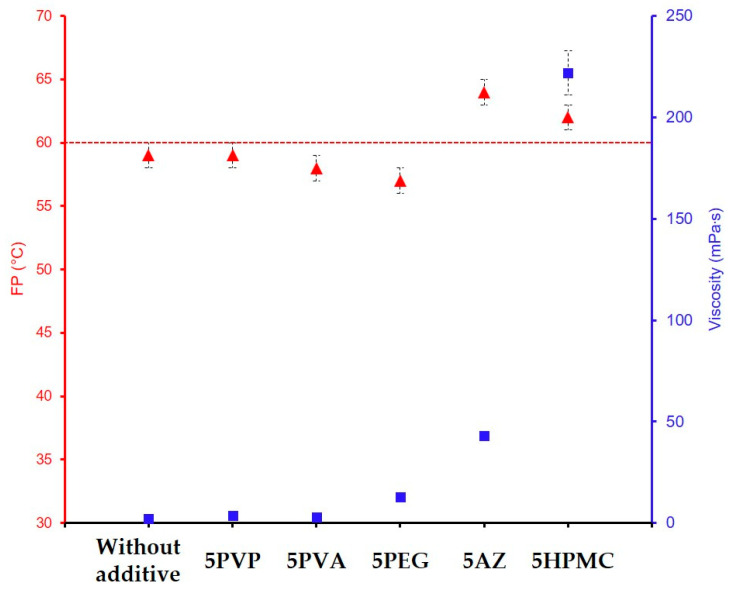
Evolution of the FP (▲) and viscosity (■) for the formulations GZ_75_-90F3 with organic additives at 5 wt%.

The flash point and the viscosity for each sol were measured for all these formulations. Ethylene glycol is the only additive introduced at 20 and 30%. These values are significantly higher than those of the other additives (1, 2, and 5%). This is due to the molecular nature of the EG additive, compared to the polymeric nature of PVA, PEG, PVP, HPMC, or AZ. For GZ_75_-90F3-20EG and GZ_75_-90F3-30EG, the results indicate an increase in the flash point (62 and 66 °C, respectively) but no effect on the viscosity of the sol while AZ and HPMC significantly increase the viscosity. The difference in chemical nature may explain why the effect is significant on the flash point due to the high boiling point of EG and why it is very low on the viscosity due to its chemical nature, which does not impact the sol–gel network. The formulation with a 5 wt% of polymeric additives is compared and presented in the Figure 9. The FP characterized for the sols with the additives PVP (59 °C), PVA (58 °C), and PEG (57 °C) are similar compared to the formulation without additives (59 °C) and there is no effect on the sol’s viscosity. These additives are not efficient for the effect expected. For the AZ and HPMC, there is an impact on the sol viscosity and the FP. For 5 wt% of AZ, the viscosity is up to 43 mPa·s with a FP of 64 °C. The same conclusion is obtained with HPMC, and a viscosity of 222 mPa·s for 5 wt% is measured. The FP exceeds 60 °C with a measured value of 62 °C. AZ et HPMC are the organic additives that allow to increase the FP above 60 °C, but at a mass percentage of five, the viscosities are slightly higher.

**Figure 10:**
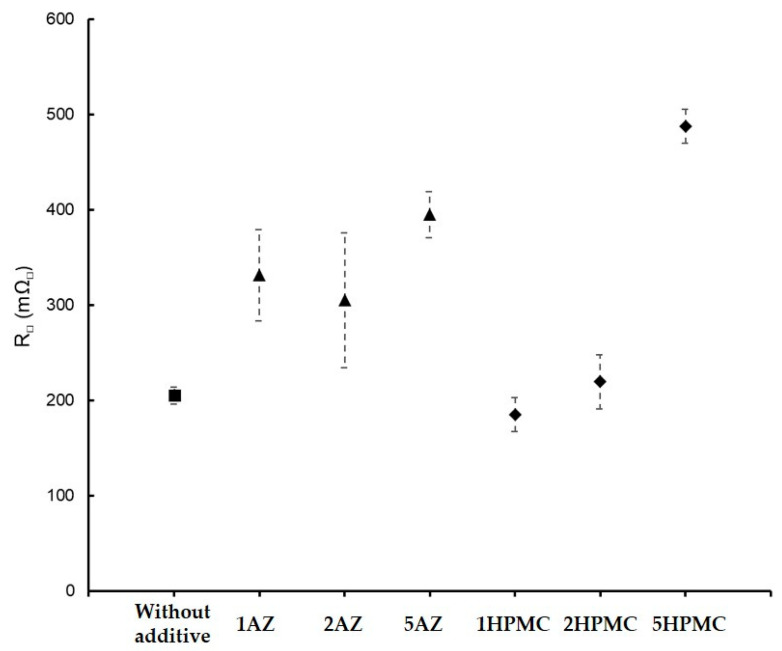
Electrical resistance evolution as a function of the additives wt% for the coatings GZ_75_-90F3: without additive (■), with AZ (▲), and with HPMC (♦).

Electrical performance was also characterized for the main promising coatings containing the organic additives AZ and HPMC with the formulation GZ_75_-90F3. The results are represented in Figure 10 for the coatings with a mass variation in AZ and HPMC. Without an additive, the electrical resistance is around 205 mΩ_□_. With AZ, the resistances are around 300 mΩ_□_ with a maximum of 687 mΩ_□_ for 3 wt%. There is not a real impact on the evolution of the electrical performance. For HPMC, an increase in the electrical resistance is visible as the function of the mass percentage. For 1 wt%, the resistance is of 185 mΩ_□_ and for 5 wt% of 486 mΩ_□_. This increase can be attributed to the very long molecular chain of the HPMC and its influence on the sol viscosity, which induced a higher probability of depositing an insulating part. The AZ and HPMC seem to have the best effect on the FP for a minimum addition and not disrupt the electrical conduction.

**Figure 11:**
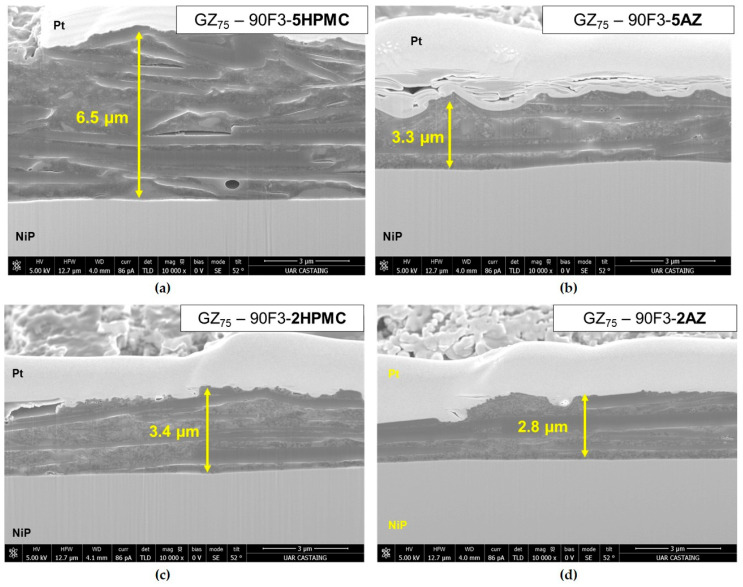
SEM pictures in cross-section after FIB preparation for the coatings (**a**) GZ_75_-90F3-5HPMC, (**b**) GZ_75_-90F3-5AZ, (**c**) GZ_75_-90F3-2HPMC and (**d**) GZ_75_-90F3-2AZ deposited at 200 mm min^−1^.

They also had an impact on the sol viscosity, which induced an improvement in the properties of the coatings because of an increase in the coating’s thickness. The coatings are homogeneous coverings and with no powdering aspect. With AZ, they are dimly grey, and with HPMC, they are entirely black. The microstructures of the coatings obtained from the sols GZ_75_-90F3-5HPMC and GZ_75_-90F3-5AZ are represented after FIB preparation in Figure 11a,b, respectively. The coatings are 6.5 and 3.3 µm thick, respectively. A similar microstructure is observed with an alternating distribution of sol–gel matrix and carbon black and graphite fillers. The graphite is perpendicular to the substrate, depending on the orientation during the withdrawal. There is a microstructure like a sandwich. Also, no porosity is identified in the area analyzed. The defects visible on the coating with 5 wt%. HPMC are induced under the electronic beam, with degradation of the organic part. This structure can be beneficial for corrosion resistance [17]. Indeed, the graphite organization reduces the corrosive electrolyte penetration. To confirm this, a NSS test was performed, and the coatings resist to 500 h of exposure without defect and corrosion product. In the Figure 11c,d, the images of the coatings GZ_75_-90F3 with 2 wt% of HPMC and AZ are presented.

The same observation is made for the coatings with 5 wt% of HPMC and AZ500 with a similar microstructure and fillers organization, but with a reduction in the thicknesses (6.5 µm vs. 3.4 µm for HPMC variation and 3.3 µm vs. 2.8 µm for AZ variation). This thickness variation is probably due to the difference in the sol viscosities for 5 wt% to 2 wt% in organic additives (222 mPa·s vs. 23 mPa·s for HPMC and 43 mPa·s vs. 14 mPa·s).

In Table 7, the characteristics of the best formulations and coating performances including their electrical properties and NSS resistance are summarized.

## 4. Conclusions

In this study, many parameters were studied to develop innovative multifunctional coatings able to exhibit antagonistic properties: corrosion resistance and electrical conduction. The combined study of the formulation parameters with the nature and filler concentration, the hydrolysis ratio, and the organic additive introduction allowed to identify their influence on the viscosity and the flash point of the sols. A mixture of carbon black and graphite, corresponding to the F3 suspension, allows to reach the targeted properties, in the case of m_cond_/m_insu_ > 0.9. In these conditions, the percolation threshold is reached. The variation in the ratio m_cond_/m_insu_ has no significative impact on the viscosity and the flash point. Relative to the hydrolysis ratio H, the viscosity increases when the H decreases (GZ_20_-100F3 with viscosity of 13 mPa·s vs. GZ_100_-100F3 with viscosity of 1.3 mPa·s). When the H ratio increases, the sol is diluted, and this positively impacts the flash point (H = 20 FP = 38 vs. H = 100 FP = 65). A good compromise for the viscosity and flash point was then identified for H in the range 50 to 75. To finish, organic additives were incorporated into the sol in order to increase the FP and to be conformal to the security rules in industrial buildings. This addition has a real impact on the sol properties, especially for the poly(2-ethyl-2-oxazoline) (AZ) and hydroxypropyl methylcellulose (HPMC).

For the coating performances, a mixture of carbon black and graphite brings benefit for a NSS resistance of 500 h. Indeed, the graphite can play the role of inhibitor with a reduction in the corrosive electrolyte infiltration. Furthermore, this compound is known for these hydrophobic properties [13]. The addition of organic additives improves the robustness of the coatings, the thickness is increased, and the reproducibility of the coatings performances really improved. The GZ50/75-90F3 formulations at 2% by weight up to 5% by weight in AZ or HPMC are the best compromise to consider the next step, which is the treatment of connectors exposed to a harsh environment.

## Figures and Tables

**Table 1:** Chemical composition of aluminum alloy AA6061.

Element	Al	Mg	Si	Fe	Cu	Zn	Mn	Cr	Ti
wt%	balance	0.8–1.2	0.4–0.8	0.7	0.15–0.4	0.25	0.15	0.04–0.35	0.15

**Table 2:** Organic additives for flash point management.

	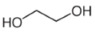	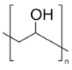	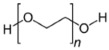	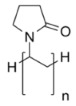	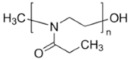	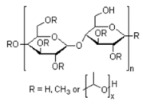
Additives ^1^	EG	PVA_13000_	PEG_1500_	PVP_3500_	AZ_500_	HPMC
Flash point (°C)	111	>113	171–235	>215	-	≥93
Boiling point (°C)	197	228	250	217	-	-

^1^ EG: ethylene glycol; PVA: polyvinyl alcool; PEG: polyethylene glycol; PVP: polyvinyl-pyrrolidone; AZ: poly(2-ethyl-2-oxazoline); HPMC: hydroxypropylmethylcellulose.

**Table 3:** Formulations based on the F1 and F2 filler suspensions.

	Formulation		Sol Characteristics			Coating Characteristics		
	m_cond_/m_insu_	H	Carbon Fillers	Viscosity (mPa·s)	Flash Point (±1 °C)	e (µm)	R_□_ (mΩ_□_)	NSS Resistance
GZ_50_-90F1	0.9	50	F1	4.0 ± 0.5	53	/	106 ± 8	500 h
GZ_50_-60F1-30F2			F1 + F2	5.0 ± 0.5	52	2 ± 0.2	109 ± 8	500 h
GZ_50_-45F1-45F2			F1 + F2	5.0 ± 0.5	53	0.7 ± 0.3	122 ± 13	500 h
GZ_50_-30F1-60F2			F1 + F2	3.0 ± 0.5	51	1 ± 0.5	134 ± 12	336 h
GZ_50_-90F2			F2	2.0 ± 0.5	52	/	1004 ± 42	336 h

**Table 4:** Formulations based on the suspension F3 with variation in the ratio m_cond_/m_insu._

Formulation	m_cond_/m_insu_	Viscosity (mPa·s)	FP (±1 °C)
GZ_50_-200F3	2	8 ± 1	55
GZ_50_-150F3	1.5	4 ± 1	52
GZ_50_-120F3	1.2	4 ± 0.5	52
GZ_50_-100F3	1	3 ± 1	52
GZ_50_-90F3	0.9	3 ± 0.5	50
GZ_50_-75F3	0.75	3 ± 0.5	48
GZ_50_-50F3	0.5	3 ± 0.5	50

**Table 5:** Characteristics of the formulations and coatings based on the F3 suspension with variation in the hydrolysis ratio H.

	Formulation		Sol’s Characteristics	
	m_cond_/m_insu_	H	Viscosity (mPa·s)	Flash Point (±1 °C)
GZ_100_-100F3	1	100	1.3 ± 0.5	65
GZ_75_-100F3		75	2 ± 0.5	58
GZ_60_-100F3		60	3.0 ± 0.4	55
GZ_50_-100F3		50	3 ± 1	52
GZ_35_-100F3		35	6 ± 1	45
GZ_20_-100F3		20	13 ± 3	38

**Table 6:** Characteristics of the formulations and coatings based on GZ_75_-90F3 formulation in presence of various organic additives.

	Formulation		Sol’s Characteristics		Coating’s Characteristics		
	m_cond_/m_insu_	H	Viscosity (mPa·s)	Flash Point (±1 °C)	e (µm)	R_□_ (mΩ_□_)	NSS Duration
GZ_75_-90F3	0.90	75	3.0 ± 0.5	59	0.3	200	500 h
GZ_75_-90F3-20EG		75	3.3 ± 0.5	62	0.1–0.3	174	1000 h
GZ_75_-90F3-30EG		75	3.3 ± 0.5	66	0.1–0.5	148	1000 h
GZ_75_-90F3-1PVP		75	3.0 ± 0.5	57	-	-	-
GZ_75_-90F3-5PVP		75	3.6 ± 0.5	59	-	-	-
GZ_75_-90F3-1PVA		75	2.0 ± 0.5	56	-	-	-
GZ_75_-90F3-5PVA		75	3.0 ± 0.5	58	-	-	-
GZ_75_-90F3-1PEG		75	4.0 ± 0.5	57	-	-	-
GZ_75_-90F3-5PEG		75	11 ± 1	57	-	-	-
GZ_75_-90F3-1AZ		75	8 ± 1	58	-	331	500 h
GZ_75_-90F3-2AZ		75	14 ± 2	59	2 ± 0.5	305	500 h
GZ_75_-90F3-5AZ		75	43 ± 2	64	3.3 ± 0.3	395	500 h
GZ_75_-90F3-1HPMC		75	10.3 ± 0.5	61	-	185	500 h
GZ_75_-90F3-2HPMC		75	23 ± 1	62	3.4 ± 0.3	231	500 h
GZ_75_-90F3-5HPMC		75	222 ± 11	63	6.6 ± 0.5	488	500 h

**Table 7:** Synopsis of the best multifunctional coatings combining anticorrosion and electrically conductive properties.

Formulations	Viscosity (mPa·s)	FP (±1 °C)	e (µm)	R_□_ (mΩ_□_)	NSS Duration
GZ_75_-90F3	3 (±0.5)	59	0.3 ± 0.1	200 ± 9	500 h
GZ_75_-90F3-2AZ	14 (±2)	59	2.8 ± 0.3	305 ± 71	500 h
GZ_75_-90F3-5AZ	43 (±2)	64	3.3 ± 0.3	395 ± 24	
GZ_75_-90F3-2HPMC	23 (±1)	62	3.4 ± 0.2	219 ± 28	
GZ_75_-90F3-5HPMC	222 (±11)	62	6.5 ± 0.3	488 ± 18	500 h

## Data Availability

The original contributions presented in this study are included in the article. Further inquiries can be directed to the corresponding author.

## Abbreviations

The following abbreviations are used in this manuscript:

m_cond_	Mass of the conductive filler
m_insu_	Mass of the sol–gel precursor constituting the insulating part of the coating
PVA	Polyvinylalcool
PEG	Polyethyleneglycol
PVP	Polyvinylpyrrolidone
AZ	Poly(2-ethyl-2-oxazoline)
HPMC	Hydroxymethylpropylcellulose
NSS	Neutral salt spray
